# Scalp Electroencephalogram-Derived Involvement Indexes during a Working Memory Task Performed by Patients with Epilepsy

**DOI:** 10.3390/s24144679

**Published:** 2024-07-18

**Authors:** Erica Iammarino, Ilaria Marcantoni, Agnese Sbrollini, MHD Jafar Mortada, Micaela Morettini, Laura Burattini

**Affiliations:** Department of Information Engineering, Engineering Faculty, Università Politecnica delle Marche, 60131 Ancona, Italy; e.iammarino@staff.univpm.it (E.I.); i.marcantoni@staff.univpm.it (I.M.); a.sbrollini@staff.univpm.it (A.S.); m.j.mortada@pm.univpm.it (M.J.M.); m.morettini@univpm.it (M.M.)

**Keywords:** brain rhythms, alpha rhythm, beta rhythm, gamma rhythm, delta rhythm, theta rhythm, engagement, working memory, epilepsy

## Abstract

Electroencephalography (EEG) wearable devices are particularly suitable for monitoring a subject’s engagement while performing daily cognitive tasks. EEG information provided by wearable devices varies with the location of the electrodes, the suitable location of which can be obtained using standard multi-channel EEG recorders. Cognitive engagement can be assessed during working memory (WM) tasks, testing the mental ability to process information over a short period of time. WM could be impaired in patients with epilepsy. This study aims to evaluate the cognitive engagement of nine patients with epilepsy, coming from a public dataset by Boran et al., during a verbal WM task and to identify the most suitable location of the electrodes for this purpose. Cognitive engagement was evaluated by computing 37 engagement indexes based on the ratio of two or more EEG rhythms assessed by their spectral power. Results show that involvement index trends follow changes in cognitive engagement elicited by the WM task, and, overall, most changes appear most pronounced in the frontal regions, as observed in healthy subjects. Therefore, involvement indexes can reflect cognitive status changes, and frontal regions seem to be the ones to focus on when designing a wearable mental involvement monitoring EEG system, both in physiological and epileptic conditions.

## 1. Introduction

Among non-invasive neuroimaging techniques, electroencephalography (EEG) is one of the most frequently used to assess the activity of the brain, allowing us to map the location and timing of brain activity and to understand how different brain regions are involved in cognitive processes [1]. The widespread use of EEG is also related to its good portability and cost-effectiveness. Moreover, recent advances in miniaturization and wireless technology have led to the development of wearable EEG systems suitable for monitoring the activity of the brain outside clinical settings, i.e., in real-life scenarios [1,2,3]. The development of such portable systems made it necessary to use the minimum number of channels as possible in order to reduce system power consumption and enhance user comfort. A critical aspect of reducing the number of electrodes to be placed is the selection of the electrodes to keep, since the electrode location determines the brain region (and consequently, the brain activity) that is registered. Indeed, since cortical brain regions have specific functions, the information provided by EEG data varies according to the area covered by the electrode [4,5,6]. In standardized systems, electrodes are uniquely marked by alphanumeric identifiers reflecting their specific location on the scalp, so that the identification of the electrode corresponds to the brain region covered. Understanding which electrodes (i.e., in standardized systems, which brain regions) have to be considered helps in designing wearable devices with the minimum number of electrodes optimized for the specific application, such as emotion recognition or cognitive monitoring. 

Including electrodes from both the left and right hemispheres is crucial due to brain lateralization, which is the tendency for certain cognitive processes to be more dominant in one hemisphere of the brain than in the other. In this context, previous studies have demonstrated that the two brain hemispheres do not equally contribute to working memory (WM), which is the ability to encode, keep, and manipulate information in the mind over a short period of time [7,8,9,10]. Specifically, Nagel et al. observed that, in adolescence, verbal WM would be lateralized to the left hemisphere while spatial WM would be lateralized to the right hemisphere, particularly in frontal brain regions [7], while Othman and colleagues confirmed that nonverbal auditory WM would be lateralized to the right hemisphere [8].

WM is often conceptualized as a mental workspace where information is actively processed. Being one of the main components of information processing, it plays a crucial role in several cognitive tasks, such as language comprehension, mathematical reasoning, decision-making, spatial processing, learning, and planning [9,10]. Given its role, WM involves the coordinated activity of multiple brain regions and neural networks, as shown non-invasively with EEG and functional magnetic resonance imaging (fMRI). Evidence from fMRI studies demonstrates that WM relies on oscillatory interactions within and between large-scale functional networks. The most involved is the frontoparietal central executive network, which interacts with other networks during WM tasks, such as the salience network and the default mode network. From EEG studies, it has been observed that neural oscillations assume specific roles in WM tasks according to their frequency. Indeed, based on the EEG frequency range (0.1–100 Hz), five EEG frequency bands, also known as EEG rhythms, can be derived: delta, theta, alpha, beta, and gamma. These rhythms, distributed along the EEG frequency range, are widely recognized, even if the frequency thresholds of each band are not uniquely defined. Among the EEG rhythms, theta and alpha are the most studied rhythms during WM tasks, but gamma rhythm, sometimes coupled with theta or alpha, has also been evaluated [11,12]. Specifically, several studies have reported theta power increases with greater levels of mental effort in frontal brain regions [10], and theta power decreases in the dorsolateral prefrontal cortex, which seems to have a crucial role for information maintenance [13]. The alpha power, instead, is often seen to decrease under conditions of greater mental effort, especially in posterior brain regions [13,14], although cases in which cognitive tasks elicit increases in alpha power have been reported as well [11,13]. In addition, sustained gamma activity has been reported during retention of several types of stimuli (visual, auditory, visuospatial, etc.) [12]. Along with EEG rhythms, the level of mental involvement in a cognitive task can also be evaluated by computing involvement (or engagement) indexes based on the spectral power of EEG rhythms, as reviewed by Marcantoni et al. [15].

WM impairment is well documented in both children and adults with epilepsy. Indeed, it is a common comorbidity of epilepsy [12,16]. It has been observed in generalized epilepsy [17], as well as in temporal lobe epilepsy [18,19], and frontal lobe epilepsy [20]. Many factors can contribute to WM impairment in epilepsy, including epileptogenic substrate, recurrent seizures, interictal epileptic activity, and anti-epileptic drugs. Given the multifactorial causes, there is an unmet need to better understand WM impairment in order to develop treatments targeting WM function in individuals with epilepsy [12].

In this context, the aim of the present study is twofold: (1) to evaluate if scalp EEG-derived involvement indexes change over time in a population of patients with epilepsy during a WM task reflecting the cognitive engagement elicited by all its phases; and (2) if so, to assess in which brain regions scalp EEG-derived involvement indexes change the most, in order to investigate whether cognitive monitoring is feasible with the use of an EEG wearable device with a reduced number of non-invasive electrodes.

## 2. Related Works

To provide a conceptual basis for understanding the objective of the present study, a literature overhaul of peer-reviewed scientific studies was carried out in Scopus in May 2024. The literature search strategy was created to cover three areas of interest for the paper: (1) the cognitive task examined here, (2) the pathology considered here, and (3) the use of EEG to investigate the functional brain state of the considered patients during the performance of the task examined, including multimodal neuroimaging studies involving EEG and fMRI. Only recent (published in the last 5 years) articles written in English were included. The articles resulting from the automatic literature search carried out in Scopus were screened with the aim of including only studies that involved spectral analysis of EEG for the assessment and characterization of brain cortical activity during WM tasks. After screening, a total of 10 articles resulted. Of these, 1 corresponds to the reference article for the real epileptic population here analyzed (described in Section 3.1) [21], whose data were originally collected to show direct evidence of hippocampal involvement in WM [22]. Thus, eventually, 9 articles were included [12,23,24,25,26,27,28,29,30]. One of them was a review article [12], while the others were original research articles [23,24,25,26,27,28,29,30]. Information referring to the original research articles that was considered relevant for this study is collected and reported in Table 1. Information includes the dimension of the population considered in the article, the type of epilepsy affecting it, the WM task examined, and the data acquired during the WM task and then analyzed. Referring to WM tasks, the visual one tests the patient’s ability to visually perceive an image or an object; the classic Sternberg one tests the patient’s ability to memorize a set of items, keep them in mind for a period of time, and recognize if the item serving as a probe was present in the set or not; modified versions of the Sternberg task were also introduced. Referring to the data, half of the original research articles exploited invasive techniques, while among the non-invasive techniques exploited by the others, one used multimodal neuroimaging, combining EEG and fMRI. 

From studies that considered fMRI data, it has emerged that WM tasks elicit activations in the frontoparietal network, and deactivations in the default mode network, central executive network, and in the visual, auditory and motor areas for visual WM tasks [12,24]. Compared to healthy controls, patients with epilepsy showed wider activations areas while deactivation areas were greatly reduced. Thus, these findings demonstrated an imbalance in the interactions between activation and deactivation networks during WM processing, which may indicate the pathophysiological mechanism of WM dysfunction in epilepsy [24]. Among the studies that considered EEG data (both scalp and intracranial one), none of them evaluated involvement indexes during a WM task. Indeed, all studies evaluated EEG rhythms, by computing the power spectral density (PSD) of the EEG frequency range of interest.

From all studies that considered EEG data, it has emerged that EEG rhythms are modulated by WM tasks, i.e., they change during the WM task according to its phases. Specifically, studies performed by Pan et al. [23,26,29] recognized theta rhythm as the most prominent EEG rhythm and frontal regions as the most prominent brain regions during the WM task. Indeed, they pointed out that the PSD of theta rhythm was higher than the PSD of all other rhythms, especially in frontal brain regions where such increases were statistically higher than in parietal and temporal brain regions. Moreover, among frontal EEG channels, theta power was higher in Fz than in all other channels. Studies [24,25,30] also confirmed the frontal region as the one showing the highest level of neural activation. Lee and coworkers also found an increase in frontal delta power and parietal alpha power during the phases of the WM task, while Dimakopoulos et al. found alpha and beta activity in temporal and occipital brain regions [25,27]. Compared to healthy controls, all studies demonstrated that patients with epilepsy showed a decreased theta power in both the frontal and occipital brain regions, especially during the encoding and retention phases of the WM task. The study performed by Liu and coworkers, instead, included an electrical stimulation in the patients before the WM task and reported that the electrical stimulation significantly increased the PSD of gamma rhythm, causing an improvement in WM performance [28].

## 3. Materials and Methods

### 3.1. Description of the Real Epileptic Population

The population analyzed in the present study comes from a public dataset published by Boran et al. in 2019 that can be downloaded at https://doi.gin.g-node.org/10.12751/g-node.d76994/ (accessed on 16 February 2024) [21]. It includes scalp EEG data recorded with the NicoletOne system (0.3–100 Hz passband, Natus^®^, https://neuro.natus.com accessed on 16 February 2024) from a population of 9 patients with epilepsy affected by drug-resistant focal epilepsy. The sampling frequency of the NicoletOne system was 256 Hz. However, scalp EEG data provided by the dataset were already resampled at 200 Hz. Scalp EEG electrodes were placed according to the 10–20 system, and the number of EEG channels recorded was not the same for all patients [21]. Details about patients’ age, gender, underlying pathology, and EEG channels recorded (derived from [21]) are reported in Table 2. 

Scalp EEG data were recorded while the patients were performing a verbal WM task which included 50 trials of about 8 s each. Thus, the database included 50 EEG signals (about 8-s long) for each patient. The WM task used was a modified Sternberg task, where encoding of memory items, retention, and recall were temporally separated. In particular, each trial started with a 1-s period in which a fixation dot was displayed on the screen (fixation period). After that, a set of consonants (stimulus) was presented for 2 s at the center of the screen (encoding period), and patients were required to retain it in memory for 3 s (retention period). All stimuli contained 8 consonants. Of these, the middle 4, 6 or 8 letters were the memory items. When the size of the memory set was 4 or 6, the outer positions were filled with ‘X’, which was never a memory item. Thus, the physical size and the visual content of the stimulus were always the same, independently from the size of the memory set. After the 3-s retention period, the probe letter was displayed on the screen and patients were asked to indicate whether the probe letter was part of the stimulus by pressing a button on a joystick (recall period). After the response, the probe was turned off and patients received acoustic feedback whether their response was correct or incorrect. Trials with different sizes of the memory set were presented in random order [10,21].

### 3.2. Computation of Brain Rhythms and Involvement Indexes

**Figure 1:**
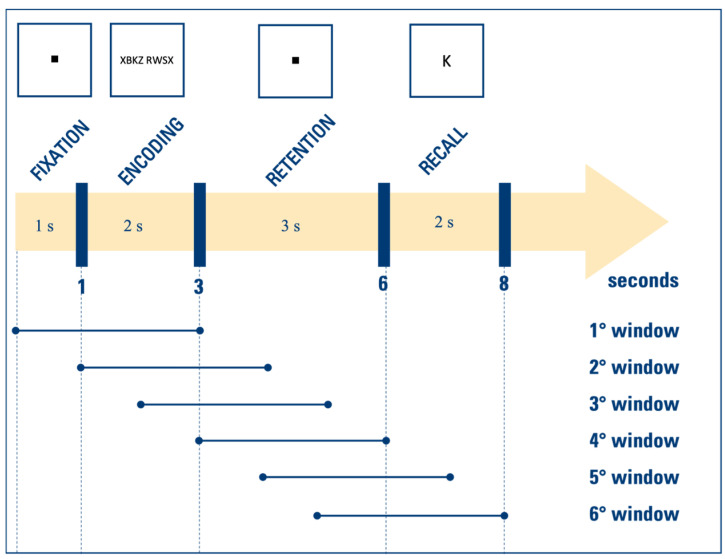
Description of the EEG windows extracted in relation to the phases of the WM task: in the upper part, the figure represents the timeline of the protocol, according to which the extraction of the EEG windows, represented in the lower part, was performed. The protocol of EEG acquisition consists of 4 consecutive periods: the fixation period lasting 1 s, the encoding period lasting 2 s, the retention period lasting 3 s, and the recall period lasting 2 s. Since the EEG windows are 3 s in duration and extracted recursively every second, the first window includes both the fixation and encoding periods, the second and third windows include both the encoding and retention periods, the fourth window covers the entire retention period, and the fifth and sixth windows include both the retention and recall periods.

EEG signals were analyzed in MATLAB R2022b using EEGLAB. The preprocessing included band-pass filtering between 0.5 Hz and 100 Hz, baseline removal, powerline removal using the EEGLAB plugin *CleanLine*, and noise removal by independent component analysis (ICA). This last preprocessing phase was performed by exploiting a specific plugin of EEGLAB, called *ICLabel*, that provided an estimation of the probability that the extracted independent components belonged to 6 possible classes [31]. The considered classes depended on the physiological or extra-physiological source contributing to the components and were: “brain”, “muscle”, “eye”, “heart”, for the physiological sources; “line noise”, “channel noise”, for extra-physiological sources; “other”, for unrecognized sources. The probability that a component belonged to a given class was automatically computed by a pre-trained classifier implemented within the plugin. Then, based on these probabilities, components were removed from the EEG signal if the percentage of “brain” source was lower than 10% or if the percentage of at least another class (i.e., “muscle”, “eye”, “heart”, “line noise”, “channel noise”, “other”) was higher than 90%. After that, for each patient, EEG signals of all trials were averaged channel by channel, and the mean EEG signal obtained was recursively windowed. Specifically, 3-s EEG windows were extracted every second until the end of the signal was reached. Figure 1 shows how the EEG windows were defined and which phases of the WM task (fixation, encoding, retention, recall) they include. 

For each EEG channel and window, EEG rhythms were extracted by applying a 6th-order bidirectional Butterworth filter. Specifically, the filter passing band was defined in the frequency range 0.5–4 Hz for delta rhythm (δ), 4–7 Hz for theta rhythm (θ), 8–12 Hz for alpha rhythm (α), 13–30 Hz for beta rhythm (β), 30–90 Hz for gamma rhythm (γ), and 12–15 Hz for somatosensory rhythm (SMR). After that, the PSD of each extracted EEG rhythm was computed for each EEG window and channel using the Welch’s overlapped segment averaging estimator. Then, among the different EEG channels, only those channels that were consistently available across all patients were further analyzed singularly to allow for comparison. The area under the PSD of each extracted EEG rhythm was computed for each EEG window and referred to as spectral power energy. Then, 37 involvement indexes were computed for each EEG window and for each patient. All indexes were spectral ratio indexes, meaning they were defined as the ratio of the spectral power energy of two or more EEG rhythms. Such indexes were identified in the systematic review published by Marcantoni et al. [15] as the most used to assess mental involvement. Their actual mathematical definition is reported in Table 3 [15].

### 3.3. Statistics

When considering the spectral power energy of the extracted EEG rhythms, overall evaluations were performed. Thus, the median spectral power energy of each extracted EEG rhythm was computed over all patients for each EEG window. When considering the involvement indexes, instead, overall evaluations were performed only for patients having the same kind of underlying pathology. Specifically, indexes of those patients were averaged (median) channel by channel. Moreover, values of each involvement index were normalized by the maximum value reached (by the same index) over the EEG windows considered. Eventually, distributions of the 37 involvement indexes were averaged over EEG channels belonging to the same brain region and paired comparisons between the first (including fixation and encoding) and the fourth window (including retention), the first and the last window (including retention and recall), the fourth and the last window were performed using the Wilcoxon signed rank test, setting the statistical significance (*p*) to 0.05. 

## 4. Results

After ICA, ICs were extracted in different number based on the number of EEG channels acquired. Overall, a maximum of four ICs out of the eight extracted, twelve ICs out of the nineteen extracted, one IC out of the ten extracted, and fifteen ICs out of the twenty extracted were identified as non-brain components and were removed from the EEG. Among the different EEG channels, six EEG channels were available in all patients; specifically, they were F3 and F4 (frontal region), C3 and C4 (central region), and O1 and O2 (occipital region). Thus, all results were computed considering these channels only. 

**Figure 2:**
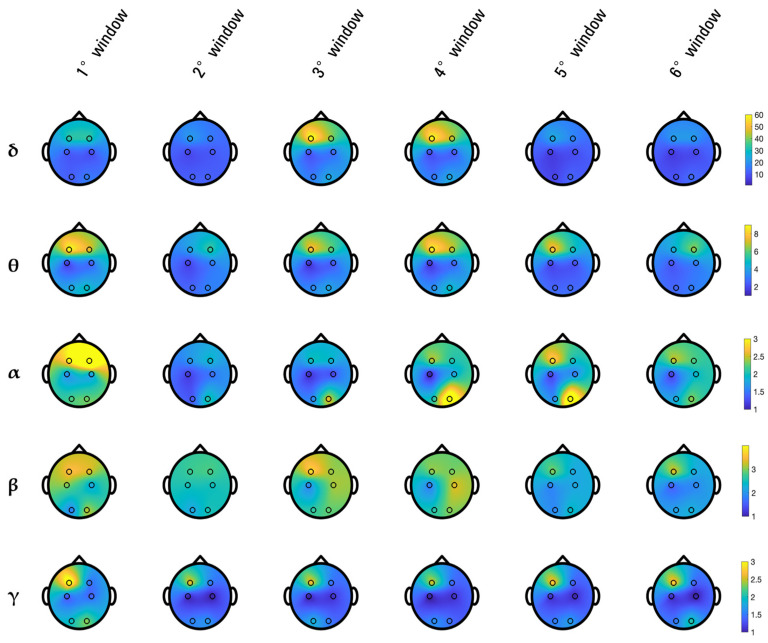
Median spectral power energy of extracted EEG rhythms.

After EEG windowing, six EEG windows were analyzed for each patient. Thus, each involvement index was described by six consecutive values, computed considering the spectral power energy of EEG rhythms in the consecutive EEG windows. The median spectral power energy of the extracted EEG rhythms (i.e., *δ*, *θ*, *α*, *β*, *γ*) computed over all patients is displayed in Figure 2 for each EEG window. Specifically, each row refers to one of the extracted EEG rhythms, and each column corresponds to an EEG window. Overall, the PSD of the delta rhythm, followed by the theta rhythm, was higher than the PSD of all other rhythms. The delta rhythm appears to increase, particularly in the frontal regions, during the retention period. The theta rhythm appears to be predominant at the beginning of the task in the frontal regions, then decreases and subsequently increases in the frontal regions during the retention period as the delta rhythm. The alpha rhythm appears to be predominant at the beginning of the task in the frontal regions, then tends to decrease and subsequently increases in the occipital regions (more on the right side), especially during the retention period. The beta rhythm appears to be predominant at the beginning of the task, then decreases and subsequently increases, especially during the retention period. The beta rhythm is less localized in a specific region than the other rhythms. Lastly, the gamma rhythm is predominant in the frontal region (more on the left) throughout the whole test.

**Figure 3:**
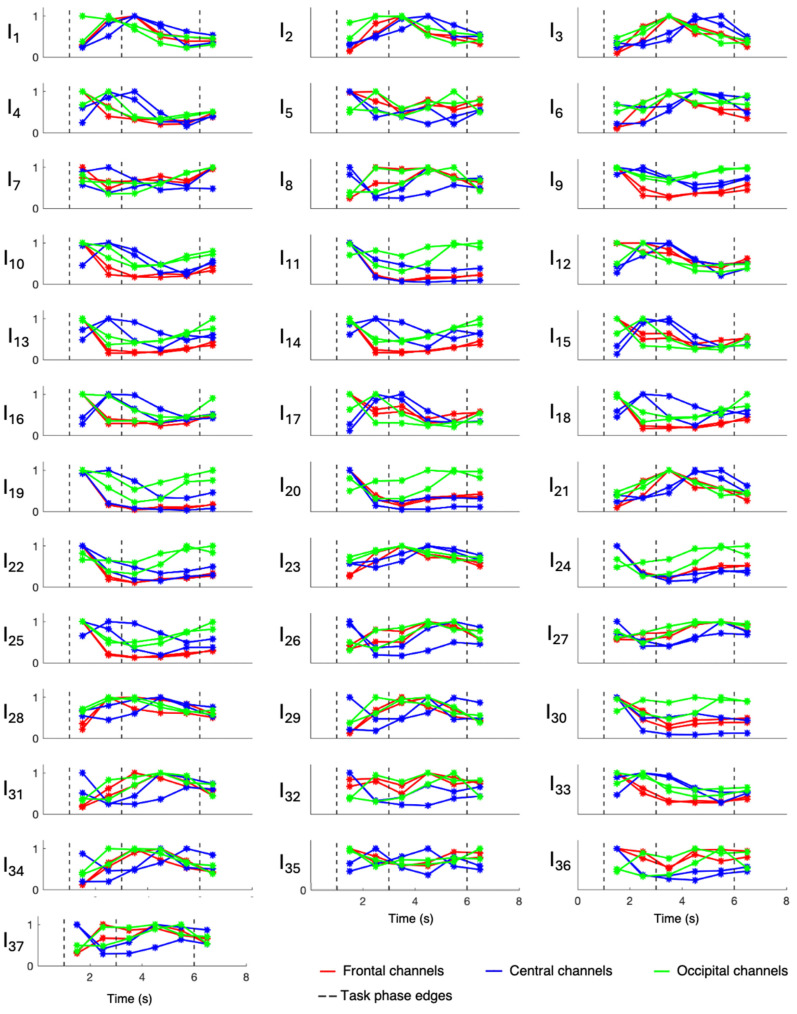
Involvement indexes over time related to patient 1 (axes limits were reported only on the first panel of each row/last panel of each column, referring to the entire row/column). The figure is divided into 37 panels, one per involvement index. Each panel contains six colored lines, one per EEG channel. Red lines refer to frontal channels (F3 and F4), blue lines refer to central channels (C3 and C4), and green lines refer to occipital channels (O1 and O2). Each line represents the trend of the involvement index over the time instants considered, since it connects the values (star markers) assumed by the index in the recursively extracted EEG windows. Dotted lines delineate the different phases of the WM task.

**Figure 4:**
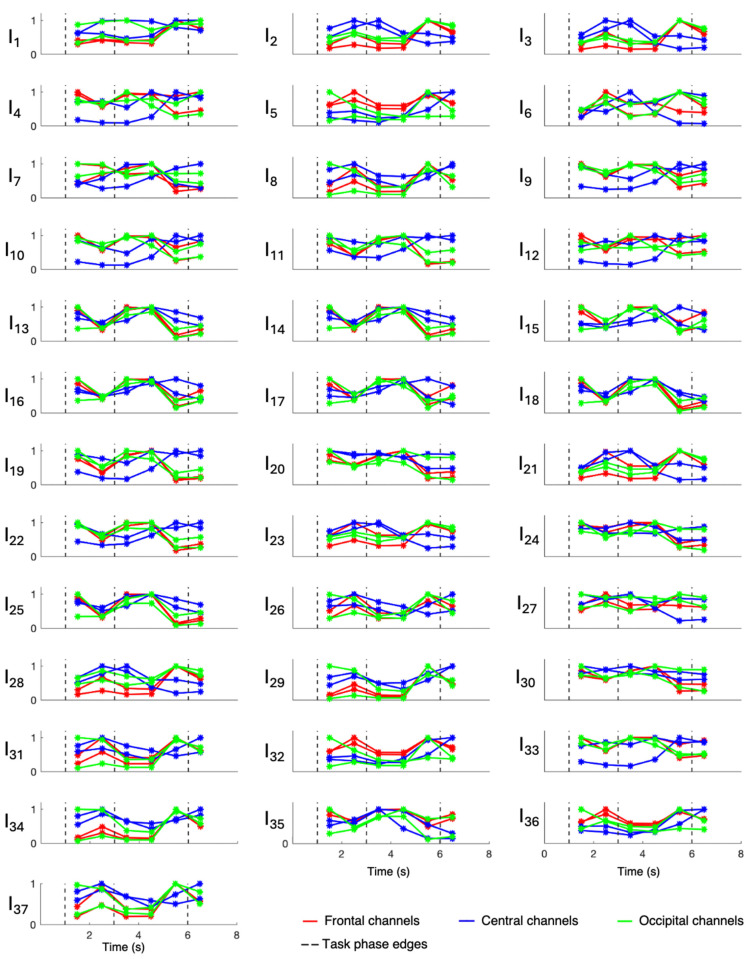
Involvement indexes over time related to patient 2 (axes limits were reported only on the first panel of each row/last panel of each column, referring to the entire row/column). The figure is divided into 37 panels, one per involvement index. Each panel contains six colored lines, one per EEG channel. Red lines refer to frontal channels (F3 and F4), blue lines refer to central channels (C3 and C4), and green lines refer to occipital channels (O1 and O2). Each line represents the trend of the involvement index over the time instants considered, since it connects the values (star markers) assumed by the index in the recursively extracted EEG windows. Dotted lines delineate the different phases of the WM task.

**Figure 5:**
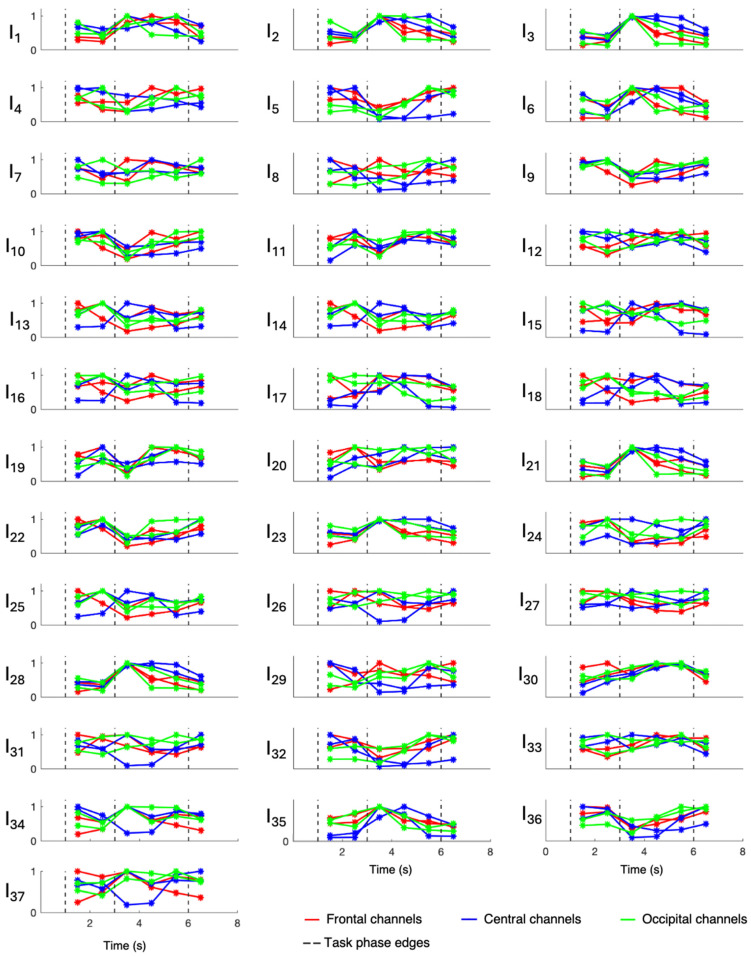
Involvement indexes over time related to patient 4 (axes limits were reported only on the first panel of each row/last panel of each column, referring to the entire row/column). The figure is divided into 37 panels, one per involvement index. Each panel contains six colored lines, one per EEG channel. Red lines refer to frontal channels (F3 and F4), blue lines refer to central channels (C3 and C4), and green lines refer to occipital channels (O1 and O2). Each line represents the trend of the involvement index over the time instants considered, since it connects the values (star markers) assumed by the index in the recursively extracted EEG windows. Dotted lines delineate the different phases of the WM task.

**Figure 6:**
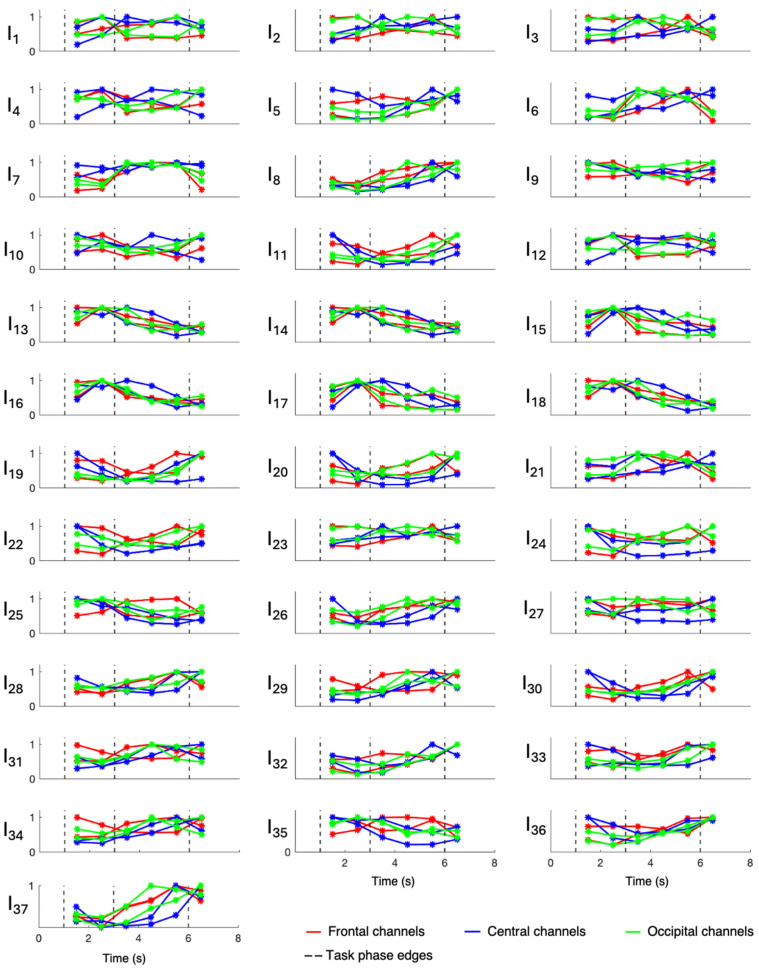
Involvement indexes over time related to patient 5 (axes limits were reported only on the first panel of each row/last panel of each column, referring to the entire row/column). The figure is divided into 37 panels, one per involvement index. Each panel contains six colored lines, one per EEG channel. Red lines refer to frontal channels (F3 and F4), blue lines refer to central channels (C3 and C4), and green lines refer to occipital channels (O1 and O2). Each line represents the trend of the involvement index over the time instants considered, since it connects the values (star markers) assumed by the index in the recursively extracted EEG windows. Dotted lines delineate the different phases of the WM task.

**Figure 7:**
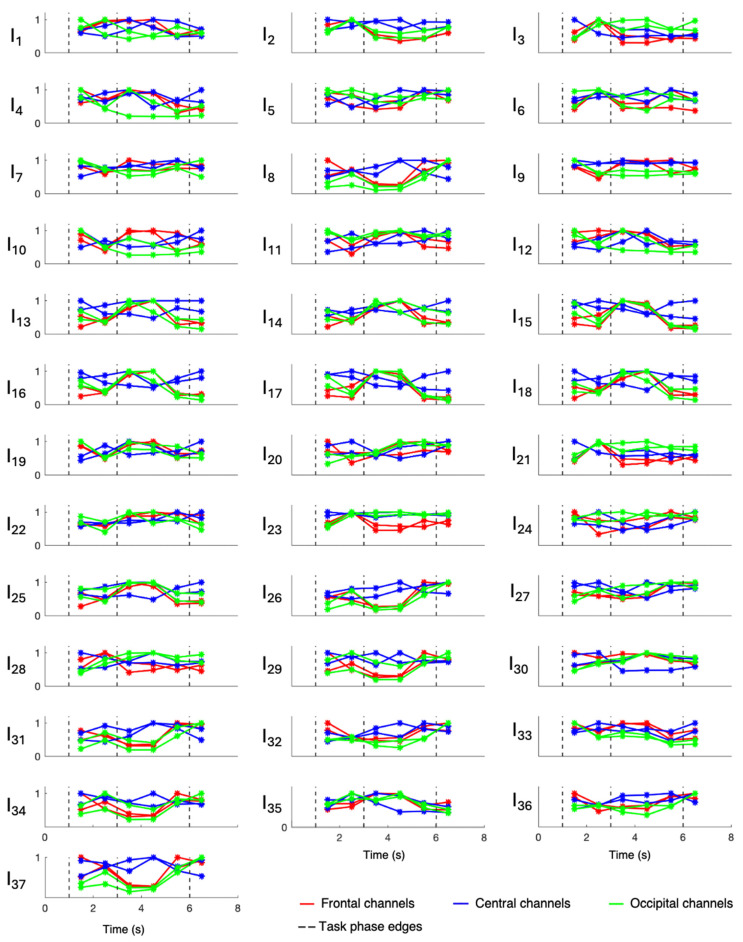
Involvement indexes over time related to the overall evaluation of patients 3, 6, 7, 8, and 9 (axes limits were reported only on the first panel of each row/last panel of each column, referring to the entire row/column). The figure is divided into 37 panels, one per involvement index. Each panel contains six colored lines, one per EEG channel. Red lines refer to frontal channels (F3 and F4), blue lines refer to central channels (C3 and C4), and green lines refer to occipital channels (O1 and O2). Each line represents the trend of the involvement index over the time instants considered, since it connects the values (star markers) assumed by the index in the recursively extracted EEG windows. Dotted lines delineate the different phases of the WM task.

Overall evaluations of involvement indexes were performed only on patients 3, 6, 7, 8, and 9, who were all affected by hippocampal sclerosis, while patients 1, 2, 4, and 5 were evaluated singularly since they were affected by different underlying pathologies. Results relative to patients 1, 2, 4, and 5 are displayed in Figure 3, Figure 4, Figure 5, and Figure 6, respectively, while those related to the overall evaluation of patients 3, 6, 7, 8, and 9 are displayed in Figure 7. In each figure, lines represent the trend of involvement indexes over the time instants considered, connecting the values assumed in the recursively extracted EEG windows. Such values are represented with a star marker placed at the central time instants of the considered windows and refer to different phases of the WM task, which are confined by dotted lines in each panel. A minimum or a maximum in the index trend indicates that the index changes over time during the WM task, while a flat index trend indicates minor changes during the WM task. The line color varies according to the cortical region covered by the EEG channels considered; specifically, F3 and F4 were represented in red, C3 and C4 were represented in blue, and O1 and O2 were represented in green. Overall, most involvement indexes had the tendency to increase during the WM task. However, indexes I_4_, I_9_–I_20_, I_22_, I_25_, I_33_, and I_35_ decreased their values with respect to the first EEG window (first star marker), especially in frontal brain regions. From Figure 7, it can be noticed that in EEG channels located in the central region, some indexes, in particular I_9_ and I_23_, do not change during the WM task. Results about the statistical difference in index distributions, possibly confirming which changes were meaningful, are shown in Table 4.

## 5. Discussion

This study evaluated whether, and in which brain regions, scalp EEG-derived involvement indexes change over time during a WM task in an epileptic population, to assess not only whether this type of monitoring is feasible but also whether it is feasible with the use of an EEG wearable device with a reduced number of non-invasive electrodes. 

To reach this aim, the dataset of Boran et al., containing simultaneous scalp EEG and intracranial EEG recordings during a verbal WM task performed by an epileptic population, was considered. This dataset was suitable for a comparison of EEG-derived involvement indexes during the different phases of the WM task. Differently from the original study (whose aim was to evaluate hippocampal involvement in WM, also analyzing the data acquired invasively) [22], the present study took into account only scalp EEG data since we were interested in testing the feasibility of involvement-index-based monitoring through wearable devices, but further studies may investigate also intracranial EEG-derived involvement indexes for comparative analysis. The choice of this dataset was justified by the fact that the protocol applied (the modified Sternberg task) was strictly timed and involved the repetition of the same task many times (50). This enabled us to assume that the cortical responses were systematically elicited (disregarding the fatigue factor), allowing us to have a common task-dependent pattern among the trials that could be extracted and highlighted by computing the mean. Indeed, according to the literature covering the analysis of EEG data obtained during a Sternberg task [13,32,33], the standard procedure involves averaging across trials of the same task. This approach assumes that all or most trials represent a single activity mode that is time-locked to the task-related events of interest while being influenced by artifactual and/or task-independent EEG activity. The mean should reveal only the systematic task-dependent EEG activity, averaging out the rest. However, task-independent activity may not necessarily be completely out of interest; rather, the resulting non-artefactual trial-by-trial variability may represent complex brain dynamic features underlying different cognitive processes [13,32,33]. Nevertheless, in the case of single-trial analysis, without averaging across multiple trials, the effect of eliminating potential artifacts or interferences is inevitably lost. Consequently, to perform this kind of trial-by-trial analysis, intracranial EEG data, which, albeit invasive, are less affected by artifacts, are preferable for a more precise analysis of neural dynamics. Therefore, the database we selected for this study, including intracranial EEG recordings, may be considered for future studies to evaluate single task-related intracranial EEG, potentially providing further insights into the dynamics of cognitive tasks and better differentiating between task-related activity and task-independent activity.

Given the different underlying pathologies affecting the participants, patients 1, 2, 4, and 5 were considered singularly, since the analysis could have different outcomes according to the underlying pathology. Moreover, given the different number of EEG channels acquired, only common EEG channels were analyzed for the computation of involvement indexes. 

EEG signals were preprocessed using EEGLAB, and artifacts were removed by ICA. ICs found by ICA decomposition were automatically classified by the recently introduced *ICLabel* plugin. This choice was prompted by the possibility of providing a systematic, objective method applicable to future studies involving larger databases. Indeed, ICs can be manually inspected and classified, but this procedure requires time, practice, and experience; instead, automated IC classifiers, such as the one used for the present study, although possibly affected by implicit classification errors, can be used to automatically classify ICs, allowing to ensure reproducibility, to avoid subjectivity, and to speed up the identification of artefactual components in cases with large populations [31].

Overlapping EEG windows lasting 3 s each were recursively extracted every second. These implementation choices represented a good trade-off among the considerations of the overall duration of the test (8 s) and of the different phases of the test, the possibility to analyze all EEG rhythms, and the possibility to obtain a quite continuous trend while referring to non-instantaneous cerebral conditions. Our approach provides insights into how EEG-derived involvement indexes can indicate transitions between different cognitive demands. Moreover, it allowed us to isolate the retention period specifically. This isolation facilitated a focused comparison between the EEG window including only this crucial phase, in which the stimulus is retained in memory, and the EEG windows including only the other periods of the task (Table 4).

Low-frequency rhythms (δ, θ) have an analogous behavior matching the information derived from the literature review carried out at the beginning of the study. These rhythms are known to be associated with cognitive processing dealing with retrieval of memory-related associative experiences, deep elaboration, and consolidation. The alpha rhythm appears to respond to the inhibition-timing hypothesis, according to which brain regions not directly involved in the cognitive task are suppressed in favor of task-related ones [13,34]. Indeed, the alpha rhythm results are more emphasized in task-irrelevant brain regions, which, in this study, were the occipital ones. High-frequency rhythms (β, γ) were found to be active throughout the entire WM task, during which the patients are cognitively engaged with a very reduced initial resting state (fixation period). Moreover, these rhythms show predominance on the left side, in accordance with Nagel et al., who observed a lateralization engaging the left hemisphere in the case of verbal WM [7].

Figure 3, Figure 4, Figure 5, Figure 6 and Figure 7 show that involvement indexes change throughout the WM task, reflecting EEG-rhythm changes, which are dependent on the cognitive engagement elicited by the different phases of the WM task. Thus, they confirmed to be indexes of cognitive engagement and showed also to be able to recognize the different levels and natures of cognitive engagement. Moreover, the index trend could be increasing, decreasing, presenting a peak, or even being flat, according to the power of the EEG rhythms from which the index is computed. In turn, this power is modulated by the cognitive status that the rhythms locally reflect in the brain region considered.

Involvement index changes were most heightened in the frontal regions, where most differences between index values over time were found to be statistically significant. Along with the frontal regions, the occipital regions also showed some significant involvement index changes. Indeed, despite the inherent subjective nature of mental processes, which may result in the activation of different brain regions from subject to subject, the literature has widely recognized the frontal and occipital regions as physiologically involved in boosting analytical reasoning and decision-making (activation or suppression) [12]. These mechanisms may be different in pathophysiological conditions like epilepsy, which could imply cognitive dysfunctions reflecting on the EEG. However, in the population here considered, only the patients affected by brain contusion and focal cortical dysplasia seem to deviate from this physiological paradigm. Thus, under both physiological and pathological conditions, with the aim of selecting the optimal electrodes to design a wearable mental involvement monitoring EEG system to be used in real-life scenarios, the frontal and occipital regions should be focused on, possibly using one electrode on the frontal region and one on the occipital region for each hemisphere, in consideration of brain lateralization.

Based on our literature review, there are no studies evaluating time changes in EEG-derived involvement indexes during a WM task, but simply time changes in single EEG rhythms. Thus, only a qualitative comparison with the literature was possible and was performed as an additional remark. It would be interesting to also consider a healthy population and a pathologic population with different conditions, because this would allow to emphasize any possible difference that could be attributed only to the epileptic condition. Nevertheless, this would imply the availability of databases in which all participants (healthy subjects and patients) are performing a WM task with the same protocol. As far as we know, these databases are not available; thus, a real statistical and quantitative inter-population comparison was not feasible for our results. 

Some limitations of the present study must be considered. Concerning the database, the main drawbacks are as follows:

- The small dimension of the population, as the database contained data from only nine patients;
- The non-uniformity of the database, since the population consists of epileptic patients with different pathological conditions (specifically xanthoastrocytoma WHO II, gliosis, hippocampal sclerosis, brain contusion, and focal cortical dysplasia, with only patients 3, 6, 7, 8, and 9 having the same pathology);
- The different number of EEG channels acquired;
- The non-availability of healthy controls performing the same task protocol as the epileptic population;
- The very short duration of the initial resting period, which prevents it from being analyzed on its own and from being considered as a reference cognitive resting status with which we could compare the other periods of the WM task.

Therefore, this study has to be stated as the observation of a small cohort of patients with epilepsy without the purpose of generalizing the obtained outcomes, but rather of providing first insights about the potential use of involvement indexes for further research. Indeed, the limited dimension of the population and the lack of a homogeneous clinical profile prevented statistically significant evaluations. Future works should perform an analogous analysis of the involvement indexes on larger databases in order to possibly confirm our preliminary results and benefit from them as a valid term of comparison.

## 6. Conclusions

This study suggests the following: (1) involvement indexes are able to reflect changes in cognitive engagement during WM tasks in the case of epilepsy, as well as in the case of using a reduced number of non-invasive electrodes, like the ones possibly designed in an EEG wearable device; (2) the frontal brain region is the cortical area mostly involved in cognitive tasks, in accordance with what was already observed in healthy subjects, even if some results seemed to show a deviation from the physiological condition. 

Therefore, monitoring cognitive engagement through involvement indexes computed from EEG acquired by a wearable device with a reduced number of electrodes appears to be feasible.

## Figures and Tables

**Table 1:** Information about the populations and the data analyzed by the included original research articles.

Reference	Pop. Size ^1^	Type of Epilepsy	WM Task	Analyzed Data
[23]	30	TLE ^2^	Visual	Scalp EEG
[24]	32	IGE ^3^	Visual	Scalp EEG and fMRI
[25]	12	NA ^4^	Verbal	Intracranial EEG
[26]	52	TLE	Visual	Scalp EEG
[27]	15	drug-resistant FE ^5^	Modified Sternberg	Intracranial EEG
[28]	8	TLE	Visual	Intracranial EEG
[29]	52	TLE	Visual	Scalp EEG
[30]	3	NA	Sternberg	Intracranial EEG

^1^ Pop. Size: population size; ^2^ TLE: temporal lobe epilepsy; ^3^ IGE: idiopathic generalized epilepsy; ^4^ NA: not available; ^5^ FE: focal epilepsy.

**Table 2:** Characteristics of the study population.

Patient	Age (Years)	Gender	Underlying Pathology	EEG Channels Recorded
1	24	Female	Xanthoastrocytoma WHO II	F3, F4, C3, C4, P3, P4, O1, O2, F7, F8, T3, T4, T5, T6, Fz, Cz, Pz, A1, A2
2	39	Male	Gliosis	F3, F4, C3, C4, O1, O2, A1, A2
3	18	Female	Hippocampal sclerosis	F3, F4, C3, C4, O1, O2, A1, A2
4	28	Male	Brain contusion	F3, F4, C3, C4, P3, P4, O1, O2, F7, F8, T3, T4, T5, T6, Fz, Cz, Pz, A1, A2
5	20	Female	Focal cortical dysplasia	Fp1, Fp2, F3, F4, C3, C4, P3, P4, O1, O2, F7, F8, T4, T5, T6, Fz, Cz, Pz, A1, A2
6	31	Male	Hippocampal sclerosis	Fp1, Fp2, F3, F4, C3, C4, P3, P4, O1, O2
7	47	Male	Hippocampal sclerosis	F3, F4, C3, C4, O1, O2, A1, A2
8	56	Female	Hippocampal sclerosis	F3, F4, C3, C4, P3, P4, O1, O2, F7, F8, T3, T4, T5, T6, Fz, Cz, Pz, A1, A2
9	19	Female	Hippocampal sclerosis	F3, F4, C3, C4, O1, O2, A1, A2

**Table 3:** Mathematical definition of the 37 involvement indexes computed.

Index	Formula	Index	Formula	Index	Formula
**I_1_**	$\frac{\beta }{\alpha }$	**I_14_**	$\frac{\delta +\theta +\alpha }{\beta }$	**I_27_**	$\frac{\alpha }{\theta +\alpha +\beta }$
**I_2_**	$\frac{\beta }{\theta +\alpha }$	**I_15_**	$\frac{\delta +\theta }{\alpha }$	**I_28_**	$\frac{\beta }{\theta +\gamma }$
**I_3_**	$\frac{\beta }{\theta }$	**I_16_**	$\frac{\delta +\theta }{\alpha +\beta }$	**I_29_**	$\frac{\beta +\gamma }{\delta }$
**I_4_**	$\frac{\theta }{\alpha }$	**I_17_**	$\frac{\delta }{\alpha }$	**I_30_**	$\frac{\alpha +\beta }{\gamma }$
**I_5_**	$\frac{\theta }{\delta }$	**I_18_**	$\frac{\delta }{\beta }$	**I_31_**	$\frac{\alpha +\gamma }{\delta +\theta }$
**I_6_**	$\frac{SMR}{\theta }$	**I_19_**	$\frac{\theta }{\gamma }$	**I_32_**	$\frac{\theta +\alpha }{\delta }$
**I_7_**	$\frac{SMR}{\beta }$	**I_20_**	$\frac{\alpha }{\gamma }$	**I_33_**	$\frac{\theta +\beta }{\alpha +\gamma }$
**I_8_**	$\frac{\alpha +\beta }{\delta }$	**I_21_**	$\frac{SMR+\beta }{\theta }$	**I_34_**	$\frac{\beta +\gamma }{\delta +\theta }$
**I_9_**	$\frac{\theta +\alpha }{\alpha +\beta }$	**I_22_**	$\frac{\theta +\alpha }{\beta +\gamma }$	**I_35_**	$\frac{\delta +\alpha }{\theta +\gamma }$
**I_10_**	$\frac{\theta }{\alpha +\beta }$	**I_23_**	$\frac{\alpha +\beta }{\theta +\alpha }$	**I_36_**	$\frac{\theta +\alpha }{\delta +\beta +\gamma }$
**I_11_**	$\frac{\theta +\alpha }{\gamma }$	**I_24_**	$\frac{\alpha }{\beta +\gamma }$	**I_37_**	$\frac{\alpha +\beta }{\delta +\theta +\gamma }$
**I_12_**	$\frac{\theta +\beta }{\alpha }$	**I_25_**	$\frac{\delta +\theta +\alpha }{\beta +\gamma }$		
**I_13_**	$\frac{\delta +\theta }{\beta }$	**I_26_**	$\frac{\alpha }{\delta +\theta +\alpha }$		

**Table 4:** Statistical differences of involvement index distributions. Each cell corresponds to a specific patient (or group of patients) and a specific brain region, indicated by the headings; pairs of EEG windows in which the resulting statistically different involvement index distributions are reported.

Patient	Frontal Region	Central Region	Occipital Region
1	All pairs	/ ^1^	4th–6th
2	All pairs	1st–4th	1st–6th; 4th–6th
4	/	1st–4th; 4th–6th	/
5	/	/	/
3, 6, 7, 8, 9	All pairs	/	1st–6th; 4th–6th

^1^ /: no pairs.

## Data Availability

The data used in the study are openly available in USZ_NCH/Human_MTL_units_scalp_EEG_and_iEEG_verbal_WM repository at https://doi.gin.g-node.org/10.12751/g-node.d76994/ (accessed on 16 February 2024).
